# Changes in Quality of Life, Adherence, and Kinesiophobia in Patients with Hemophilia Treated with Extended Half-Life Treatment: Final Results of the LongHest Project

**DOI:** 10.3390/ph17070835

**Published:** 2024-06-25

**Authors:** Roberto Ucero-Lozano, Raúl Pérez-Llanes, Rubén Cuesta-Barriuso, Elena Donoso-Úbeda

**Affiliations:** 1 Department of Physiotherapy, European University of Madrid, 28670 Madrid, Spain; roberto.ucero@universidadeuropea.es; 2 InHeFis Research Group, Instituto Asturiano de Investigación Sanitaria (ISPA), 33011 Oviedo, Spain; 3 Department of Physiotherapy, University of Murcia, 30100 Murcia, Spain; rperez@um.es; 4 Department of Surgery and Medical-Surgical Specialties, University of Oviedo, 33003 Oviedo, Spain; 5 Department of Physiotherapy, Catholic University San Antonio-UCAM, 30107 Murcia, Spain; edonoso@ucam.edu

**Keywords:** hemophilia, EHL, quality of life, adherence, kinesiophobia

## Abstract

(1) Background: Hemophilia is a bleeding disorder characterized by hemarthrosis. Prophylaxis is the gold standard for bleeding prevention. Extended half-life (EHL) recombinant FVIII replacement products have shown to be associated with low bleeding rates. The aim was to evaluate the efficacy of EHL prophylaxis in improving perceived quality of life, adherence to treatment, and kinesiophobia in patients with hemophilia. (2) Methods: This was a prospective cohort study. Forty-six patients from different regions, who had started EHL FVIII concentrate prophylactic treatment, were evaluated at baseline and at 12-month follow-up. The study variables were as follows: perceived quality of life (36-Item Short Form Health Survey), adherence to treatment (Validated Hemophilia Regimen Treatment Adherence Scale—Prophylaxis), and kinesiophobia (Tampa Scale of Kinesiophobia). (3) Results: There were statistically significant differences in the domains Role-Physical (*p* < 0.001), Bodily Pain (*p* < 0.001), Role-Emotional (*p* < 0.001), Vitality (*p* = 0.04), and Social Functioning (*p* = 0.01) and the total scores, Physical Health (*p* < 0.001) and Mental Health (*p* < 0.001) on perceived quality of life. There were significant differences in the domains Skipping (*p* < 0.01), Communicating (*p* < 0.001), and the total score (*p* = 0.01) in terms of adherence. There were also significant differences in kinesiophobia (*p* = 0.02) after the study period. (4) Conclusions: EHL prophylaxis can improve the perceived quality of life of people with hemophilia. This prophylactic regimen, which requires fewer infusions, may improve adherence to treatment in adult patients with hemophilia over a 12-month period. The administration of extended half-life factor VIII concentrates can reduce kinesiophobia in adult patients with hemophilic arthropathy.

## 1. Introduction

Hemophilia is a congenital bleeding disorder caused by a deficiency or absence of essential blood clotting proteins, namely clotting factor VIII (hemophilia A) or factor IX (hemophilia B). This deficiency leads to reduced thrombin generation [1]. Hemophilia A and B present similar symptoms and are classified according to the severity of the clotting factor deficiency: severe hemophilia when the factor level is <1%, moderate when it is 1–5%, and mild when values are 5–40% [2]. The prevalence of hemophilia A is 24.6:100,000, while the ratio is 5.0:100,000 in the case of hemophilia B [3]. 

Bleeding in the musculoskeletal system, mainly hemarthrosis, is the main clinical manifestation in people with severe hemophilia [4]. The recurrence of joint bleeds leads to the development of a hemophilic, painful, and disabling arthropathy, which very adversely affects the perceived quality of life in these patients [5]. Prophylaxis is the optimal standard of care for patients with severe hemophilia, significantly reducing bleeding episodes in these patients versus on-demand treatment subsequent to the development of a bleeding event [6]. This treatment mainly includes intravenous clotting factor replacement therapy [4]. 

Reduction of the frequency of bleeding episodes, associated with prophylactic treatment, can be translated into improved long-term joint function, a lower number of hospitalizations, and a much improved perception of health-related quality of life [7,8]. However, the regular administration of clotting factor concentrates with a short half-life poses difficulties such as adherence to treatment or venous access problems, since these patients need the regular administration of intravenous infusions. Extended half-life (EHL) recombinant FVIII (rFVIII) replacement products [9] and recombinant humanized bispecific monoclonal antibodies mimicking the function of activated FVIII have been developed in the last decade [10]. 

The therapeutic administration of EHL offers the possibility of administering flexible doses and customizing treatment to meet the needs of each patient [11]. EHL products, which feature a recombinant FVIII Fc fusion protein (rFVIIIFc), have shown low annualized bleeding rates (ABR) in patients with severe hemophilia A undergoing individualized prophylactic treatment [12,13]. Prophylactic treatment with rFVIII-Fc has shown significant improvements by reducing bleeding rates, with a higher proportion of patients without bleeding events, as well as joint health benefits, with less frequent infusions and a lower consumption of clotting factor [14]. Most of these benefits were observed both in the adult population and in children under 12 years of age, promoting a more active lifestyle in these patients. The first results of this project [15] showed improvements in the frequency of hemarthrosis and pain intensity in adult patients with severe hemophilia A on EHL treatment. 

The early administration of prophylactic treatment directly impacts how a patient manages the disease. Patients with hemophilia who receive early prophylaxis show a better perceived quality of life than those who initiate prophylaxis later on who, in addition, must cope with the physical sequelae derived from hemophilic arthropathy [7]. Adherence to treatment, on-demand or prophylaxis, significantly reduces the likelihood of suffering from major chronic pain [16].

A high degree of kinesiophobia is associated with severe pain, disability, and a poorer quality of life [17], being common in people suffering from chronic pain [18]. The risk of bleeding in people with hemophilia, and the pain secondary to bleeding, or the risk of suffering an injury, cause an increased level of kinesiophobia in this population [19,20]. Accordingly, reducing the frequency of bleeding, joint damage, and pain through prophylaxis could reduce the level of kinesiophobia of these patients.

The main objective of the study was to evaluate the effectiveness of prophylaxis with recombinant FVIII Fc fusion protein in improving the perceived quality of life in adult patients with severe hemophilia A. The secondary objective was to analyze the effectiveness of this treatment with rFVIIIFc on adherence to prophylactic treatment in these patients, as well for reducing fear of movement, after a 12-month study period.

## 2. Results

Fifty-one patients with hemophilia were invited to participate in the study. For personal reasons, two patients declined to participate, while three patients had antibodies to FVIII concentrates and failed to meet that selection criterion.

A total of 46 patients with hemophilia participated in the study. All patients enrolled in the present study followed the same therapeutic protocol with the same commercially available EHL-rFVIII (Eloctate^®^). After the 12-month follow-up period, two patients did not attend the final evaluation. The mean age of the sample was 38.62 (SD: 6.42) years. All patients presented hemophilia A with a severe phenotype of the disease. The average body mass index was 26.78 (SD: 4.26) kg/m^2^. Joint damage at the beginning of the study, was 8.47 (SD: 2.08), 7.02 (SD: 2.16) and 9.10 (SD: 2.78) points, in ankle, knee, and elbow, respectively. The descriptive characteristics of the sample are shown in Table 1.

After the study period, there were statistically significant differences (*p* < 0.001) in the variables Role-Physical (ES = 2.58), Bodily Pain (ES = 0.76), Role -Emotional (ES = 4.73), and in the domains Vitality (*p* = 0.004) and Social Functioning (*p* = 0.01). In the total scores, Physical Health (ES = 0.75) and Mental Health (ES = 1.14), there were also significant changes (*p* < 0.001) at 12-month follow-up. 

In the adherence to treatment, we found statistically significant differences in the domains Skipping (*p* = 0.002), Communicating (*p* < 0.001), and the total score (*p* = 0.01). There were significant differences in the Kinesiophobia variable (*p* = 0.02) after the study period. The statistics of central tendency and dispersion in the evaluations carried out and the observed changes are shown in Table 2.

When calculating the percentage of patients whose changes after the study period exceeded the value of the minimum detectable change, it was noted that more than half of the patients exceeded that threshold in the variables Role-Physical (91.30%), Bodily Pain (58.69%), Role-Emotional (100%), and Mental Health (84.78%). On the other hand, the percentage was 47.82% in the variables Physical Function and Physical Health total score. The statistics of the minimum detectable change are shown in Table 3.

## 3. Discussion

The aim of this study was to assess the effect of a prophylactic treatment with extended half-life clotting factor concentrates on psychosocial variables of perceived quality of life, adherence, and kinesiophobia in adult patients with severe hemophilia A. An improvement was found in their perceived quality of life in the items: role-physical, bodily pain, vitality, social functioning, role-emotional, physical health, and mental health. A lower frequency of bleeding in patients with hemophilia, of all ages, was associated with a higher quality of life of this population [21]. During the 12-month study period, there was a significant reduction in the number of bleeds with the administration of EHL [15], which would explain the changes in the physical components of the perceived quality of life (role-physical, bodily pain, and physical health). A reduced bleeding frequency in patients with prophylactic treatment is related to an improved function and quality of life in patients with hemophilia [22]. Therefore, the reduced bleeding frequency observed in the first results of this study [15] can be related to an improvement in function that allows these patients to move better. Moreover, this lower frequency of hemarthrosis in patients with hemophilia treated with EHL reduces the nociceptive source of these bleeding events. Accordingly, such reduced bleeding can be related to a lower peripheral sensitization and, with it, a lower irritability of the nervous system and a lower hyperalgesia [23]. 

There is a correlation between the pain perceived by patients with hemophilia and aspects such as vitality, social functioning, emotional role, mental health, and total physical health perceived [24]. In this project, the number of hemarthroses decreased and, therefore, pain also decreased, and the patients presented better values in the items vitality, social functioning, emotional role, total physical health, and total mental health. In addition, chronic pain can lead to sleeping disorders, depression, and fatigue, limiting activities of daily living and causing psychosocial stress [25]. Changes in patients’ pain can influence their mood and fatigue, relating to the observed changes in perceived quality of life. The percentage of subjects that exceeded the minimum detectable change was above 50% in almost all items where there were significant changes, so this can be considered as a clinically relevant improvement in these patients.

Patients on EHL prophylaxis also showed improvements in kinesiophobia. Kinesiophobia, or fear of movement, is an excessive, irrational, and debilitating fear of performing physical movement. This fear is due to a feeling of vulnerability due to painful injury or reinjury [17]. Hemarthrosis suffered by patients with hemophilia is accompanied by acute pain [26], increasing the activation of nerve fibers and contributing to tissue damage via oxidative stress that generates the release of cytokines. This leads to nociceptor activation and an exacerbation of pain [27], so a higher intensity of pain causes further fear-avoidance behaviors [28]. Accordingly, the lower frequency of hemarthrosis with EHL prophylaxis may contribute to reducing the nociceptive sources that trigger the painful experience, allowing the patient to be gradually exposed to more movement.

This study also observed changes in adherence to EHL prophylaxis, presenting improvements in the variables’ treatment compliance, communication with health professionals, and total adherence to treatment. A good adherence includes a better compliance, avoiding the loss or delay of infusions and a more stable effect. Such therapeutic stability may be related to an improvement in the frequency of hemarthrosis. Clotting factor concentrates with a short half-life require a greater number of infusions and, therefore, a greater number of actions by the patient. In this way, the number of opportunities for forgetting was higher and, with it, for the effect of the medication to be destabilized with the resulting risk of bleeding. Patients with hemophilia are used to the regular administration of treatment in a preventive or therapeutic way and to maintaining well-established medication habits. Therefore, the change in adherence observed in this study could be related to the direct effects of EHL drugs: the simplicity of administration, fewer infusions, and reduced bleeding events. 

### 3.1. Implications for Clinical Practice

The results of this study should make health systems reflect on the advantages of this type of drug among the therapeutic options for patients with hemophilia; health expenditure associated with the number of hemorrhagic events decreases in clinical practice. Therefore, it would be of interest to evaluate the economic impact of these extended-half-life drugs on the associated expenses in the short-term (minor surgical interventions, drugs, hospitalizations, etc.), and in the long-term (orthopedic replacement surgery, analgesic drugs, etc.). 

The vicious cycle represented by bleeding–synovitis–bleeding is behind the development of hemophilic arthropathy. Lowering the frequency of bleeding with EHL products [14,15] could positively change the life of people with hemophilia, being able to delay or prevent hemophilic arthropathy in pediatric or young adult patients without previous joint damage. In adult patients with signs of arthropathy, the reduction of bleeding would favor the release of iron at the joint level, the apoptosis of chondrocytes and, with this, slow down the progression of arthropathy as much as possible. This would directly delay eventual joint replacement. Ultimately, it would be of interest to compare the changes in the variables perceived quality of life and adherence to treatment between patients receiving treatment with normal half-life factor VIII concentrates and those receiving extended half-life FVIII concentrate treatment in order to conclusively compare the relationship of the drug with these variables.

### 3.2. Study Limitations

Although the sample size was adjusted to that required before the study, a broader analysis that would allow us to calculate the most determining factors of quality of life perceived by these patients was not possible. Similarly, the absence of ultrasound studies did not rule out the development of subclinical hemarthrosis during the study period. The results of our study suggest that adherence to therapy and quality of life improve during the administration of extended half-life recombinant FVIII concentrates over the study period. However, these results should be taken with caution as the results, in terms of compliance and quality of life with a prophylactic regimen based on normal half-life FVIII concentrates, are unknown.

## 4. Materials and Methods

### 4.1. Study Design

A single-blind prospective cohort study to evaluate the efficacy of prophylactic treatment with rFVIIIFc in adult patients with hemophilic arthropathy was conducted.

### 4.2. Subject Recruitment

The patients were recruited between July 2020 and September 2021. The study was developed by the Hemophilia and Physiotherapy research group (InHeFis Research Group), with the collaboration of the Spanish Hemophilia Federation and provincial associations for the recruitment of patients. The recruitment centers were the hemophilia associations of the regions of Andalusia, Aragon, Castilla y León, Galicia, Madrid, Murcia, and Valencia.

The inclusion criteria of the study were as follows: patients with hemophilia A, severe hemophilia phenotype (<1% FVIII), over 18 years of age, who had started prophylactic treatment with rFVIIIFc in the month before the start of the study, having hemophilic arthropathy in at least 3 joints, more than 3 points on the Hemophilia Joint Health Score [17], and no orthopedic surgeries scheduled during the study period.

The exclusion criteria of the study were as follows: antibodies to clotting factor concentrates (inhibitors), patients were unable to walk, and people with cognitive impairments limiting the understanding of the various assessments.

### 4.3. Ethical Consideration

The principal investigator explained to the patients the objectives of the study, and the possible risks and benefits of taking part. All patients signed the informed consent document. This study was designed in accordance with the Helsinki guidelines. 

The Research Ethics Committee of the University of Murcia approved the completion of this study (ID: 2511/2019). The research project was prospectively registered in the Protocol Registration and Results System (NCT03914209). The study was approved (S-201901700001036) by the Spanish Agency for Medicines and Other Health Products (Ministry of Health).

### 4.4. Measurement Instruments

Patients were evaluated at baseline (T0) and at 12-month follow-up (T1). The investigators travelled to all regions, and all the patients were assessed by the same assessors following the same methodology. All evaluations were performed by the same evaluator, with years of experience in the evaluation and treatment of patients with hemophilia, blinded to the study conditions. The main variable of the study was perceived quality of life. The secondary variables were adherence to treatment and kinesiophobia. 

Perceived quality of life was measured with the Spanish version of the 36-Item Short Form Health Survey [SF-36] [29]. This self-administered scale consists of 36 items, using 8 domains: physical functioning, limitations due to physical problems, physical pain, social role or function, mental health, limitation due to emotional problems, vitality, and general perception of health.

Using the Validated Hemophilia Regimen Treatment Adherence Scale—Prophylaxis [VERITAS-Pro] [30], the adherence of patients with hemophilia to prophylactic treatment was measured. This questionnaire was completed by the patients. This instrument is composed of 24 Likert-type items rated from 1 to 5 (never or 0% of the time, rarely or 25% of the time, sometimes or at least 50% of the time, often or at least 75% of the time, and always or 100% of the time) grouped in five dimensions: Timing, Dosing, Planning, Remembering, Skipping, and Communicating. The minimum adherence score was 24 and the maximum adherence score was 120; on each subscale, the score ranged between 4 and 20 points.

The patients’ fear of movement or physical activity was assessed with the Tampa Scale of Kinesiophobia [TSK] [31]. This self-administered instrument consists of 11 items, with a 4-point Likert scale. The score range is 17–68, with more than 37 points being considered a high score.

At the beginning of the study, the main variables were collected, namely, anthropometric (weight, height, and body mass index), clinical (joint damage with the Hemophilia Joint Health Score) [32], and socio-demographic (work, age, degree of sedentary lifestyle, and degree of physical activity according to the International Physical Activity Questionnaire) [33]. 

### 4.5. Sample Size

The sample size was calculated using the statistical package G*Power (version 3.1.9.2; Heinrich-Heine-Universität, Düsseldorf, Germany). Assuming a mean effect size (d = 0.60), with an alpha level (type I error) of 0.05, and a statistical power of 99% (1-β = 0.99), a sample size of 38 patients with hemophilic knee arthropathy was estimated. Given the forecast of a possible 20% of dropouts during the follow-up year, 46 adult patients with severe hemophilia A were recruited from 7 different locations.

### 4.6. Statistical Analysis

With version 19.0 of the SPSS statistical package for Windows (IBM Company, Armonk, NY, USA), statistical analyses were performed. Descriptive statistics of central tendency (mean) and dispersion (standard deviation) of the quantitative variables of the study were calculated. The changes after the study period (T1–T0) were calculated with the parametric paired samples *t*-test. The size of the effect of the changes was calculated with the G*Power program using the mean difference and the standard deviation of the difference as statistics, being classified as large (d > 0.80), medium (d > 0.50), or small (d > 0.20). The minimum detectable change (MDC) was calculated by estimating the standard error of measurement (SEM). The SEM was calculated using the formula: SEM = SD_pre_ × (√ 1 − ICC) [34]. The intraclass correlation coefficient was used as a reliability measure [35]. Based on the SEM, the MDC was obtained (MDC = Z-score × (√ 2 × SEM)). The confidence level was set at 95%. An intention-to-treat analysis was performed in this study. The level of significance of the study was α < 0.05.

## 5. Conclusions

Prophylactic treatment using extended half-life factor VIII concentrates may improve the perceived quality of life for patients with hemophilia. Prophylaxis needing fewer infusions leads to a better adherence to treatment by adults with hemophilia. The lower frequency of hemarthrosis with EHL drugs can improve the kinesiophobia of these patients, favoring their mobility and functionality. Studies that compare the results of perceived quality of life and adherence to treatment in patients receiving normal half-life versus extended half-life concentrate prophylaxis are needed.

## Figures and Tables

**Table 1:** Descriptive characteristics of the patients included in the study.

Variables	Mean (Standard Deviation)
Age (years)	38.62 (6.42)
Weight (kg)	85.43 (7.94)
Height (cm)	177.43 (4.82)
Body mass index (kg/m^2^)	26.78 (4.26)
Joint health status *	
Ankle (0–20)	8.47 (2.08)
Knee (0–20)	7.02 (2.16)
Elbow (0–20)	9.10 (2.78)
	***n* (%)**
Lifestyle **	
Sedentary (<3000 MET minutes per week)	18 (39.1)
Active (>3000 MET minutes per week)	28 (60.9)
Workstation	
Standing/moving	16 (34.77)
Sitting	18 (39.13)
Both	12 (26.08)

* Hemophilia Joint Health Score; ** International Physical Activity Questionnaire.

**Table 2:** Descriptive analysis (means and standard deviation) and changes in the variables.

Variables	Domains	T0	T1	MD (SD)	95%CI	ES
Adherence	Timing	6.57 (2.04)	6.54 (1.92)	−0.02 (0.95)	−0.30; 0.26	0.02
	Dosing	4.98 (1.77)	4.80 (1.18)	−0.17 (1.46)	−0.60; 0.26	0.12
	Planning	5.43 (2.12)	5.74 (2.24)	0.30 (1.34)	−0.09; 0.70	0.22
	Remembering	8.17 (3.53)	8.35 (3.42)	0.17 (2.11)	−0.45; 0.80	0.08
	Skipping	5.48 (1.69)	6.11 (2.01)	0.63 (1.30) *	0.24; 1.01	0.48
	Communicating	7.09 (1.97)	8.39 (2.62)	1.30 (2.19) **	0.65; 1.95	0.59
	Total score	37.72 (8.07)	39.93 (8.64)	2.21 (5.85) *	0.47; 3.95	0.38
Kinesiophobia		32.02 (5.75)	30.96 (6.37)	−1.06 (3.04) *	−1.96; −0.16	0.35
Quality of life	Physical Function	65.11 (25.48)	68.48 (23.23)	3.37 (41.28)	−8.89; 15.63	0.08
	Role-Physical	16.52 (9.57)	77.72 (28.49)	61.19 (23.63) **	54.17; 68.21	2.58
	Bodily Pain	61.13 (24.23)	74.93 (16.78)	13.80 (18.09) **	8.43; 19,17	0.76
	General Health	48.85 (24.52)	50.65 (23.45)	1.80 (9.28)	−0.95; 4.56	0.19
	Vitality	65.43 (18.55)	70.22 (19.46)	4.78 (10.59) *	1.63; 7.92	0.45
	Social Functioning	81.09 (26.99)	90.30 (15.72)	9.21 (23.20) *	2.32; 16.11	0.40
	Role-Emotional	22.46 (6.10)	94.17 (19.08)	71.71 (15.14) **	67.22; 76.21	4.73
	Mental Health	81.22 (15.79)	83.30 (14.80)	2.08 (8.46)	0.42; 4.60	0.25
	Physical Health	39.32 (9.36)	43.62 (10.82)	4.29 (5.71) **	2.59; 5.98	0.75
	Mental Health	45.95 (6.56)	54.52 (11.28)	8.57 (7.51) **	6.33; 10.80	1.14

T0: Outcome measures at baseline; T1: outcome measures at 12 months; MD: means difference; SD: standard deviation of the difference; 95%CI: 95% confidence interval; ES: effect size. * Significant difference between assessments (*p* < 0.05). ** Significant difference between assessments (*p* < 0.001).

**Table 3:** Calculation of the minimum detectable changes in the study variables.

Variables	Domains	ICC	SEM	MDC (MDCp)
Adherence	Timing	0.93	0.50	1.96 (8.69)
	Dosing	0.77	0.83	2.53 (8.69)
	Planning	0.89	0.68	2.29 (4.34)
	Remembering	0.89	1.12	2.94 (13.04)
	Skipping	0.86	0.63	2.20 (8.69)
	Communicating	0.71	1.05	2.84 (28.26)
	Total score	0.86	3.01	4.81 (21.73)
Kinesiophobia	Kinesiophobia	0.93	1.488	3.38 (13.04)
Quality of life	Physical Function	0.43	27.06	14.42 (47.82)
	Role-Physical	0.55	6.39	7.01 (91.30)
	Bodily Pain	0.76	11.67	9.46 (58.69)
	General Health	0.96	4.84	6.09 (13.04)
	Vitality	0.91	5.37	6.42 (36.95)
	Social Functioning	0.61	16.65	11.31 (26.08)
	Role-Emotional	0.60	3.85	5.44 (100)
	Mental Health	0.91	4.54	5.91 (15.21)
	Physical Health	0.91	2.74	4.59 (47.82)
	Mental Health	0.80	2.92	4.74 (84.78)

ICC: intraclass correlation coefficient; SEM: standard error of measurement; MDC: minimal detectable change; MDCp: proportion of minimum detectable change.

## Data Availability

The data that support the findings of this study are available on request from the corresponding author. The data are not publicly available due to privacy or ethical restrictions.

## Abbreviations

EHL	extended half-life
FVIII	clotting factor VIII
rFVIII	recombinant FVIII
rFVIIIFc	FVIII Fc fusion protein
ABR	annualised bleeding rate
T0	baseline
T1	12-month follow-up
SF-36	36-question short-form health survey
Veritas-Pro	Validated adherence scale for adherence to prophylactic treatment of haemophilia regimen
TSK	Tampa kinesiophobia scale
MCD	minimum detectable change
SEM	standard error of measurement
